# A Laplacian based image filtering using switching noise detector

**DOI:** 10.1186/s40064-015-0846-5

**Published:** 2015-03-08

**Authors:** Ali Ranjbaran, Anwar Hasni Abu Hassan, Mahboobe Jafarpour, Bahar Ranjbaran

**Affiliations:** School of Electrical and Electronic Engineering, Universiti Sains Malaysia, Engineering Campus, 14300 Nibong Tebal, Seberang Perai Selatan, Pulau Pinang Malaysia

**Keywords:** Local noise estimator, Denoising, Total variation, Energy functional, Laplacian

## Abstract

This paper presents a Laplacian-based image filtering method. Using a local noise estimator function in an energy functional minimizing scheme we show that Laplacian that has been known as an edge detection function can be used for noise removal applications. The algorithm can be implemented on a 3x3 window and easily tuned by number of iterations. Image denoising is simplified to the reduction of the pixels value with their related Laplacian value weighted by local noise estimator. The only parameter which controls smoothness is the number of iterations. Noise reduction quality of the introduced method is evaluated and compared with some classic algorithms like Wiener and Total Variation based filters for Gaussian noise. And also the method compared with the state-of-the-art method BM3D for some images. The algorithm appears to be easy, fast and comparable with many classic denoising algorithms for Gaussian noise.

## Introduction

Denoising is one of the most important issues in image processing. The most popular noise removal methods are Adaptive Median Filtering (AMF), Total Variation (TV) based algorithms, Kernel based methods, Bilateral and Guided filtering and recently BM3D state-of-the-art in natural image denoising. In this work, we introduce a noise removal approach using a local noise estimator. We use the noise estimator in a minimization energy functional scheme. We obtain an iterative image denoising process using Laplacian. Denoising can be seen as adding values of pixels with their relative Laplacian weighted by local noise estimator.

Section 2 is related work. Section 3 represents our idea for denoising. We show how we can define a noise estimator using the sign of change in intensity of pixels in a 3x3 window. Using local noise estimator modified by Gaussian weight, we define an energy functional, drive the final equation and use it in an iterative denoising process. We show that although Laplacian is known as edge detector, it can be used for noise removal purposes. The algorithm is implemented in section 4. In section 5, results are shown and described. Moreover, figures of the denoising method are shown in comparison with Wiener, ROF and BM3D filters based on TV value and visual performance.

## Related work

A Total Variation based noise removal method (ROF) (Rudin et al. 1992) defines an energy functional that preserves edges of the image and smoothes Gaussian noisy area, based on the total variation norm minimizer. The TV regularization technique is a suitable method that can be extended to different noisy conditions such as Laplace and Poisson (Chan and Esedo Lu 2005; Li et al. 1994). The Split Bregman method (Goldstein and Osher 2008) is fast, reliable and extendable to different models of noise distribution. Split Bregman is a basic and effective tool in solving many functional-based problems such as Compressed Sensing (CS) (Candès et al. 2006). In recent years, the usage of kernel-based techniques in image denoising has developed the quality of noise removal results. The image used in kernel functioning is called the guidance image. One of the most popular approaches using the guidance image is bilateral filter (Petschnigg et al. 2004). Other important kernel-based methods are Data-adaptive kernel regression (Takeda et al. 2007), Non-Local Means (Buades et al. 2005) and Optimal Spatial Adaptation (Kervrann et al. 2006). Another novel state of the art method was recently introduced as guided filter (He et al. 2010). The US patent 6229578 “Edge Detection Based Noise Removal Algorithm” (Acharya et al. 2001) is a denoising method based on using edge detector. This method removes noise by distinguishing between edge and non-edge pixels and applying a first noise removal technique to pixels classified as non-edge and a second noise removal technique to pixels classified as edge pixels. BM3D is a well-engineered algorithm which represents the current state-of-the-art method for denoising images corrupted by Additive White Gaussian Noise (AWGN) (Dabov et al. 2007; Dabov et al. 2006; Dabov et al. 2008; Dabov et al. 2009; Chen and Wu 2010). In another strategy, denoised image is considered as a linear combination of the original image and its average when the coefficients are determined by an edge detector (Ranjbaran et al. 2013).

## Methodology

**Figure 1:**
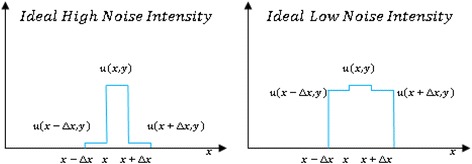
**Two ideal noisy pixels.**

Our methodology is based on using a local noise estimator in an energy functional minimizing scheme. Here we start with explaining our idea to define a local noise estimator. Consider Figure 1 where *u*(*x*, *y*) is pixel intensity. A noise in x direction is estimated when the sign of the change of the image intensity for two adjacent pixels is in opposite direction. Taking two x direction gradient components *g*_*x*_ and *g*_*x* + Δ*x*_ we write:

1 $$ {g}_x=\frac{u\left(x,y\right)-u\left(x-\varDelta x,y\right)}{\varDelta x}\kern3.5em {g}_{x+\Delta x}=\frac{u\left(x+\Delta x,y\right)-u\left(x,y\right)}{\varDelta x} $$

A noise is detected when *g*_*x*_ < 0, *g*_*x* + Δ*x*_ > 0 or *g*_*x*_ > 0, *g*_*x* + Δ*x*_ < 0. Generally we have:

$$ -{g}_x\ {g}_{x+\Delta x}>0 $$

Using Heaviside function *H* the noise detector in x direction can be defined as:

2 $$ SW{N}_x=H\left(-{g}_x\ {g}_{x+\Delta x}\right) $$

**Figure 2:**
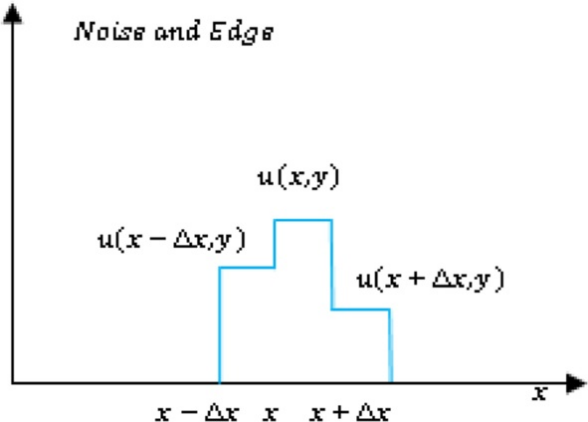
**Pixels include noise and edge.**

Noise appears in two conditions in an image, first as ideal noise, shown in Figure 1 and second as correlated noise (Figure 2). To distinguish these two cases we need to assign a weight to the detected location. Because *SWN*_*x*_ acts as a switch its value is between zero and one. For ideal noisy pixels in the image plane, independent of the noise intensity, the value of weight should be one.

For the cases where noise is added to an edge as shown in Figure 2 the weight should be lower than one. To find a measure of the weight we consider that for ideal case |*u*(*x* + *Δx*, *y*) − *u*(*x* − *Δx*, *y*)| = 0 independent of noise intensity, but for non-ideal noise this difference is not zero. Then we can use a Gaussian weighting for the detected noise as the following equation:

3 $$ W{N}_x={e}^{-{\left({g}_x+{g}_{x+\Delta x}\right)}^2} $$

Where:

4 $$ {g}_x+{g}_{x+\Delta x}=\frac{u\left(x+\varDelta x,y\right)-u\left(x-\varDelta x,y\right)}{\varDelta x} $$

As noise is added to the image in two directions we should drive the similar equations for y components. By using similar notations we find the final switching noise estimator as:

5 $$ SWN=H\left(-{g}_x\ {g}_{x+\Delta x}\right)H\left(-{g}_y\ {g}_{y+\Delta y}\right){e}^{-{\left({g}_x+{g}_{x+\Delta x}\right)}^2}{e}^{-{\left({g}_y+{g}_{y+\Delta y}\right)}^2} $$

To find a denoising way using *SWN* we define a measure of noise intensity in the image plane. Since *SWN* ≥ 0 the intensity of the noise in the noisy image can be defined as:

6 $$ {\displaystyle {\displaystyle \iint }H\left(-{g}_x\ {g}_{x+\Delta x}\right)H\left(-{g}_y\ {g}_{y+\Delta y}\right){e}^{-{\left({g}_x+{g}_{x+\Delta x}\right)}^2}{e}^{-{\left({g}_y+{g}_{y+\Delta y}\right)}^2}} $$

To reduce noise, we define the following energy functional and try to minimize it:

7 $$ J={\displaystyle \iint }{\left(u-{u}_0\right)}^2+\lambda {\displaystyle \iint }H\left(-{g}_x\ {g}_{x+\Delta x}\right)H\left(-{g}_y\ {g}_{y+\Delta y}\right){e}^{-{\left({g}_x+{g}_{x+\Delta x}\right)}^2}{e}^{-{\left({g}_y+{g}_{y+\Delta y}\right)}^2} $$

where *u* and *u*_0_ are denoised and noisy images respectively and the first part is regularization term. The energy functional is minimized in Appendix A. Although there are some techniques for computing *u* by Equ. 47, this way is a bit sophisticated and difficult to implement. To find a more simple equation we add *λ f*(*u*_0_) to the two sides of the equation and write:

8 $$ {u}_0+\lambda\ f\left({u}_0\right)=u+\lambda \left(f\left({u}_0\right)-f(u)\right) $$

Based on *SWN* operation and iteratively computing *f*(*u*_0_) and *f*(*u*) in the locations classified as noise, we approximate *f*(*u*_0_) = *f*(*u*). Then the final equation we used in our implementation is:

9 $$ u={u}_0+\lambda\ f\left({u}_0\right) $$

**Figure 3:**
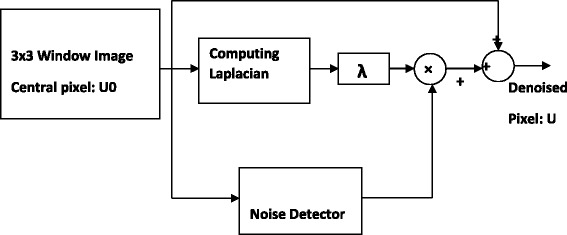
**Block diagram of the denoising method.**

This relation can be interpreted as follows: Because *u* is disturbed by *f*(*u*) and creates noisy image *u*_0_ (Equ. 47), a similar process can restore *u* from *u*_0_ (Equ. 9). By decreasing noise after some cycles of iteration *f*(*u*_0_) goes to a small value. Equ. 47 presents a noise cancellation method based on using Laplacian value. The algorithm decreases the noise by adding the pixels value with Laplacian that weighted by *SWN*. Laplacian has been known as a common second-order edge detector but it has considerable value in noisy condition. Block diagram of the method is demonstrated in Figure 3.

Adding the intensity of pixels with the relative Laplacian is an averaging process. In an iterative process we can generally consider the evolution equation as:

10 $$ u\left(t+\Delta t\right)=u(t)+\left(\lambda\ SWN\kern0.5em {\nabla}^2u\ \right)\Delta t $$

where Δ*t* is the evolution timing step. For the cases that *SWN* = 1 the evolution equation is:

11 $$ u\left(t+\Delta t\right)=\left(1-\lambda \right)u(t) + \lambda\ \left(\frac{u\left(\mathrm{t}-\Delta t\right)+u\left(\mathrm{t}+\Delta t\right)}{2}\ \right)\kern0.5em \Delta t $$

Then we have the following first order differential equation:

12 $$ \dot{u}+\lambda u(t) = \lambda\ \overline{u} $$

When $\overline{u}=\frac{u\left(\mathrm{t}-\Delta t\right)+u\left(\mathrm{t}+\Delta t\right)}{2}$ is the average value of *u*. The timing response is:

13 $$ u(t) = \overline{u}+\left({u}_0-\overline{u}\right)\ {e}^{-\lambda t}\kern2.75em t\ge 0 $$

where *λ* is the time constant. For *λ* ≥ 0 the denoised image *u* exponentially approaches to it steady state value *ū* in the locations where *SWN* is high. To find the constraints on *λ* we note that because 0 ≤ *u* ≤ 1 and − 1 ≤ *f*(*u*_0_) ≤ 1, we have two constrains on *λ* as:

14 $$ -{u}_0\le \lambda \le 1-{u}_{0\kern0.75em }\kern3.25em -\left(1-{u}_0\right)\le \lambda \le {u}_0 $$

As 0 ≤ *λ* ≤ 1 − *u*_0_ and we choose ≥ 0, maximum value for *λ* is:

15 $$ {\lambda}_{max}=1 $$

It is predictable that in implementing the algorithm by large number of iterations *λ* must be a positive small value. In real condition *SWN* is not constant and reduced during the evolution.

## Implementation

We have implemented our method in Matlab for Gaussian noise and evaluated it on different images. For implementation the Heaviside function is approximated by inverse tangent function:

16 $$ H\left(-{g}_x\ {g}_{x+\Delta x}\right)H\left(-{g}_y\ {g}_{y+\Delta y}\right)\approx \frac{\frac{\pi }{2}+ta{n}^{-1}\left(-C{g}_x{g}_{x+\Delta x}\right)}{\pi}\kern0.5em \frac{\frac{\pi }{2}+ta{n}^{-1}\left(-C{g}_y{g}_{y+\Delta y}\right)}{\pi } $$

According to Equ.12*λ* controls timing response and the value is between 0 and 1. So choosing the middle value can be suitable. Based on experimental results *C* = 3 is found as an appropriate value for noisy cases. We use a 3x3 window for simplicity and fast computing. The method is implemented by 10 numbers of iteration. The algorithm of implementation can be shown as the following steps:

1. Setting parameters : window size 3×3, *λ* = 0.5
2. Reading image *u*_0_
3. Adding zero mean Gaussian Noise (imnoise code)
4. Computing *SWN* for current pixel
  17 $$ SWN=\frac{\frac{\pi }{2}+ta{n}^{-1}\left(-3{g}_x{g}_{x+\Delta x}\right)}{\pi}\kern0.5em \frac{\frac{\pi }{2}+ta{n}^{-1}\left(-3{g}_y{g}_{y+\Delta y}\right)}{\pi }\ {e}^{-{\left({g}_x+{g}_{x+\Delta x}\right)}^2}{e}^{-{\left({g}_y+{g}_{y+\Delta y}\right)}^2} $$
5. Computing Laplacian for current pixel
  18 $$ {\nabla}^2u = \frac{u\left(x+\Delta x,y\right)+u\left(x-\Delta x,y\right)-2u\left(x,y\right)\kern0.5em }{4}+\frac{u\left(x,y+\Delta y\right)+u\left(x,y-\Delta y\right)-2u\left(x,y\right)\kern0.5em }{4} $$
6. Updating *u* = *u*_0_ + *λ SWN* ∇^2^*u*_0_ for the current pixel
7. Going to step 4 and continuing
8. Finishing when the whole of the image is scanned for ten times.

## Results and discussions

**Figure 4:**
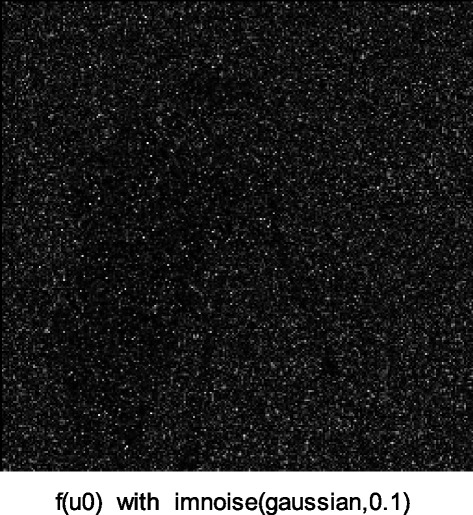
***f*** **(** ***u***_**0**_ **)** **for Cameraman with 0.1 noise variance.**

**Figure 5:**
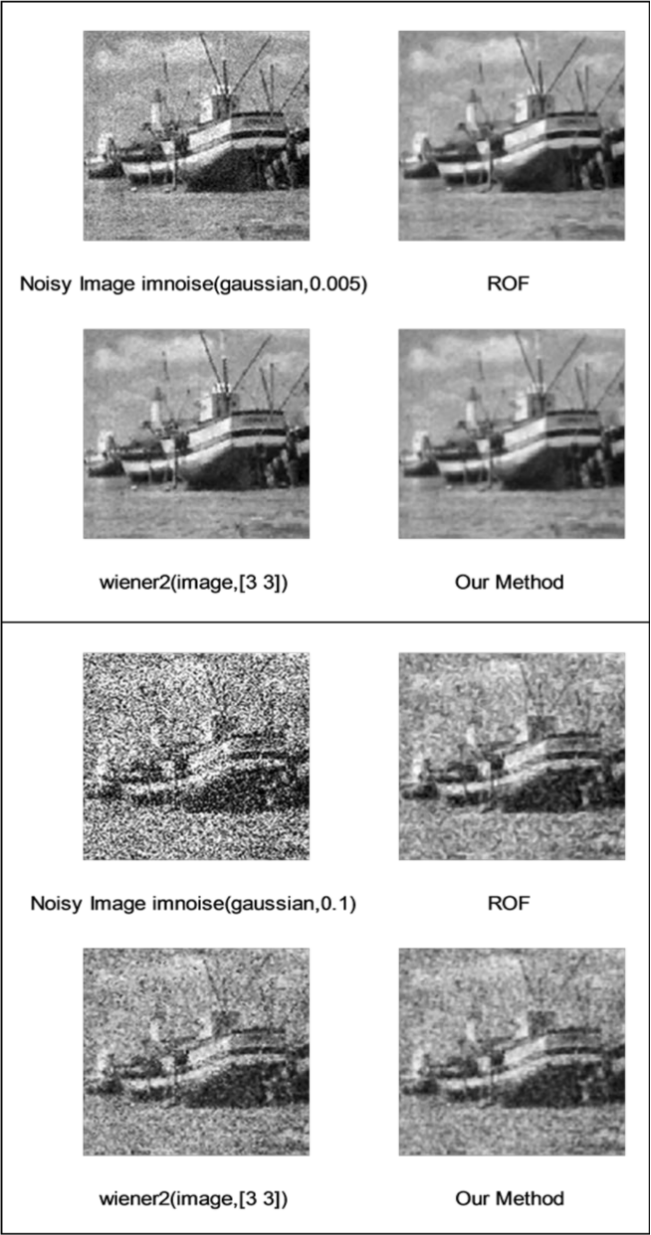
**Results of ROF, wiener filter and our denoising method for Boat.**

**Figure 6:**
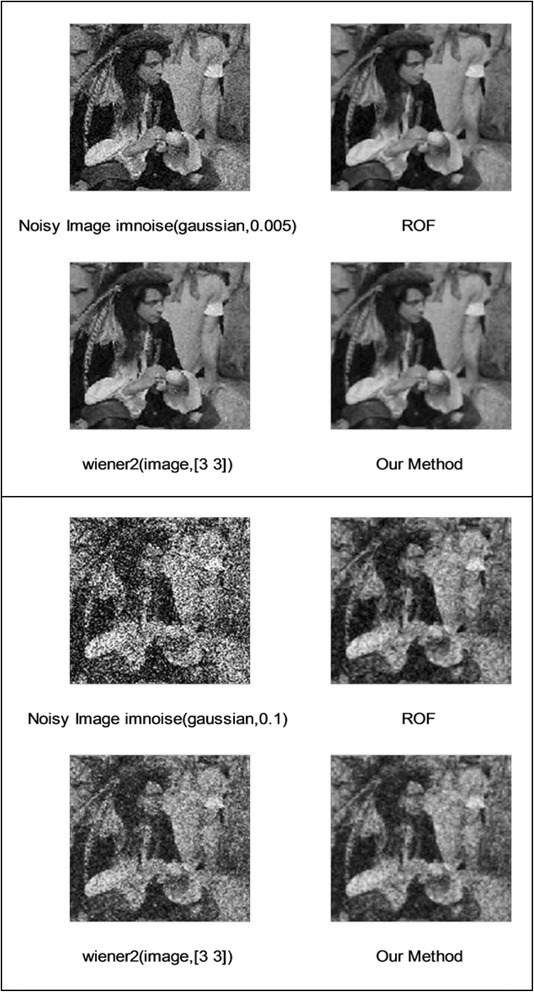
**Results of ROF, wiener filter and our denoising method for Man.**

**Figure 7:**
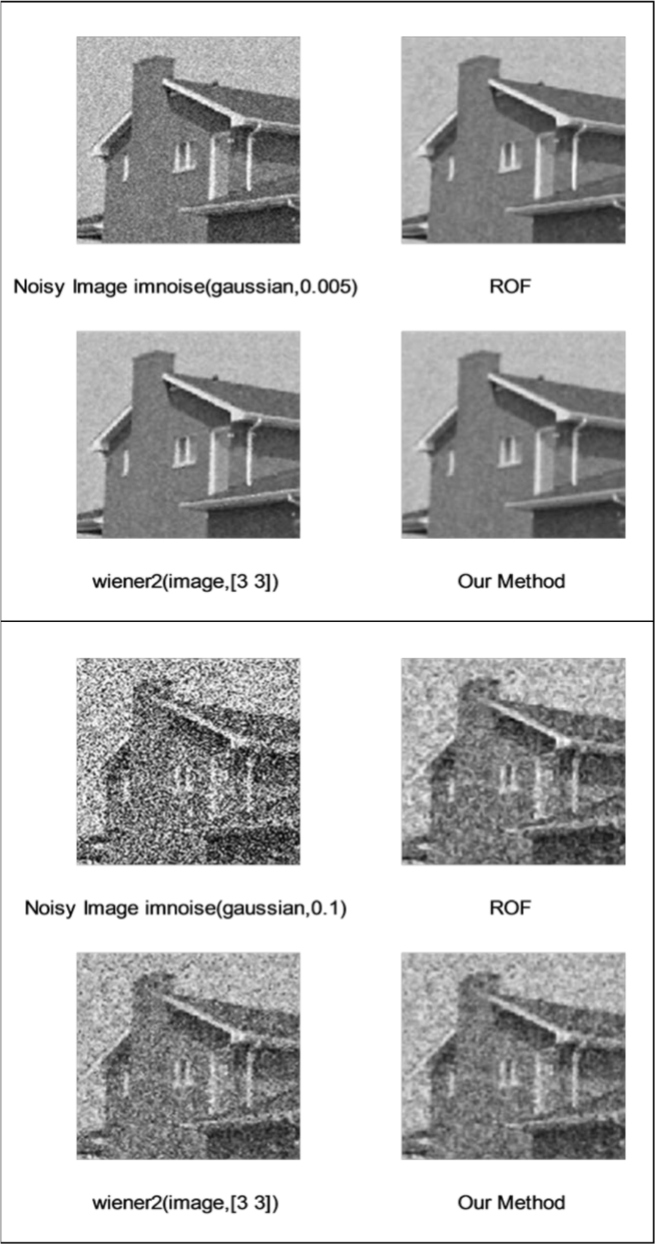
**Results of ROF, wiener filter and our denoising method for House.**

**Figure 8:**
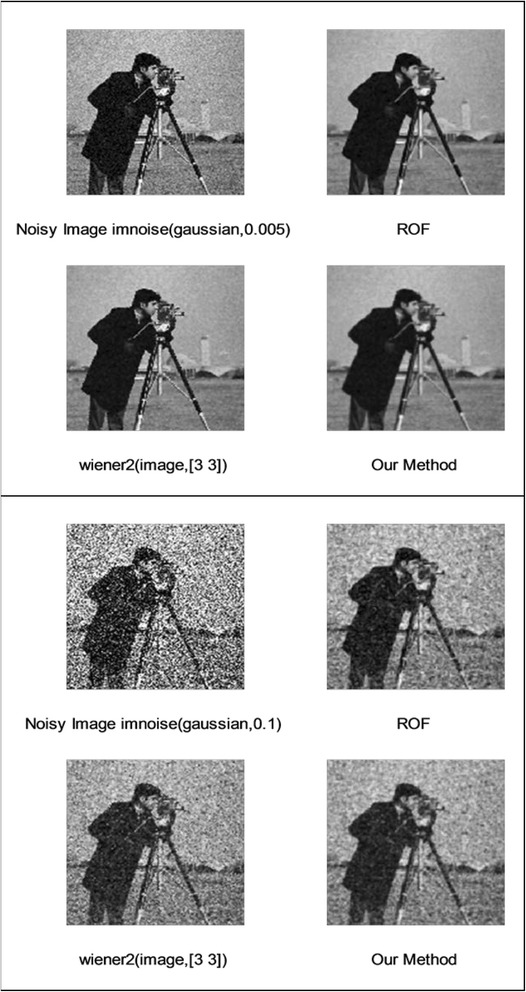
**Results of ROF, wiener filter and our denoising method for Cameraman.**

**Figure 9:**
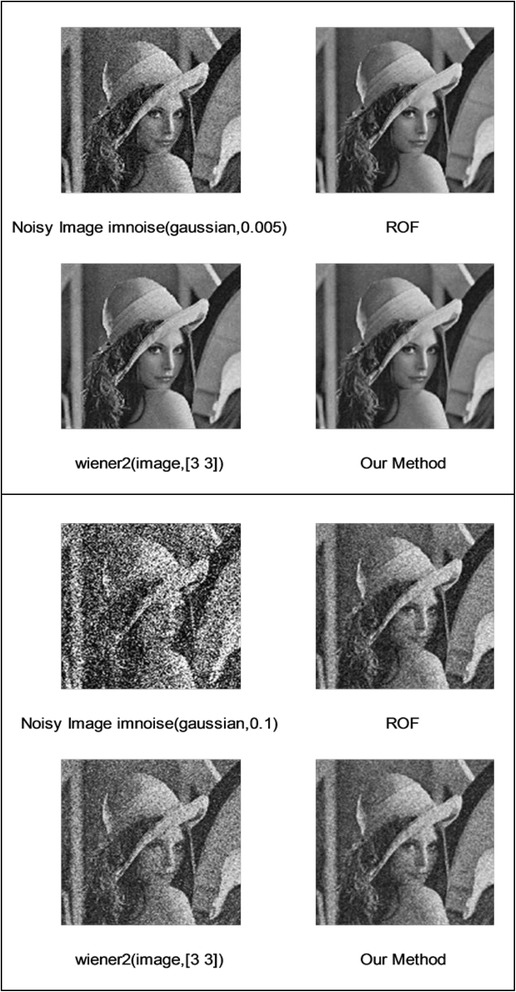
**Results of ROF, wiener filter and our denoising method for Lena.**

**Figure 10:**
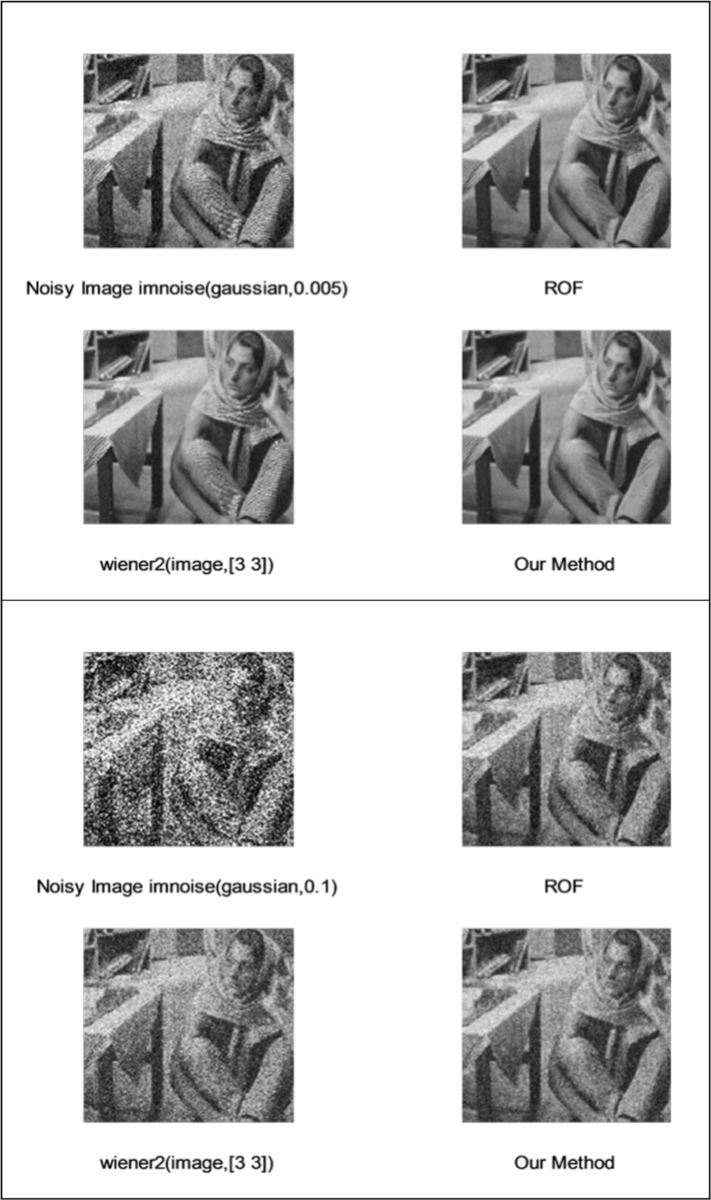
**Results of ROF, wiener filter and our denoising method for Barbara.**

**Figure 11:**
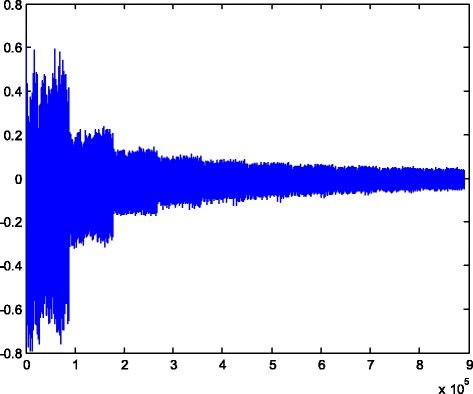
**Decreasing behavior of** ***f*** **(** ***u***_**0**_ **)** **during the denoising process.**

**Figure 12:**
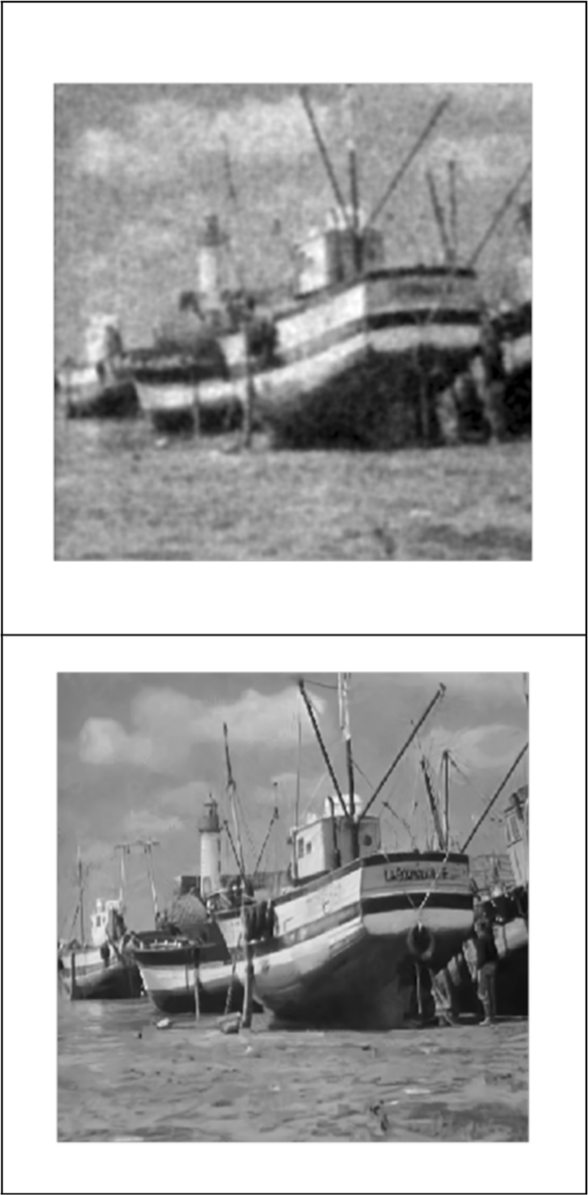
**Results of our method (up) and BM3D technique (down) with SNR = 25 for Boat.**

**Figure 13:**
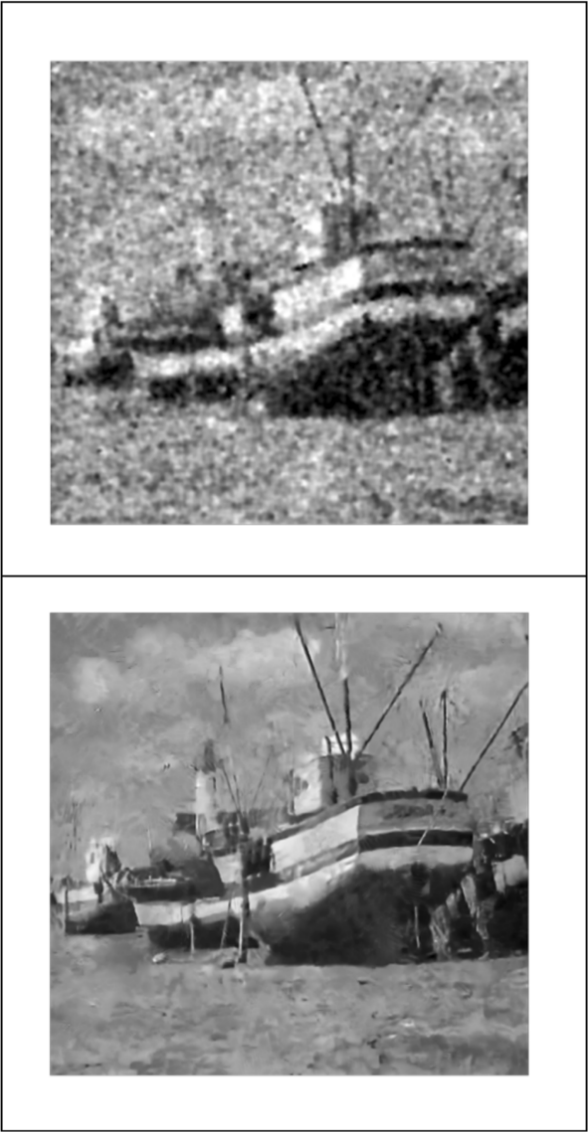
**Results of our method (up) and BM3D technique (down) with SNR = 75 for Boat.**

**Figure 14:**
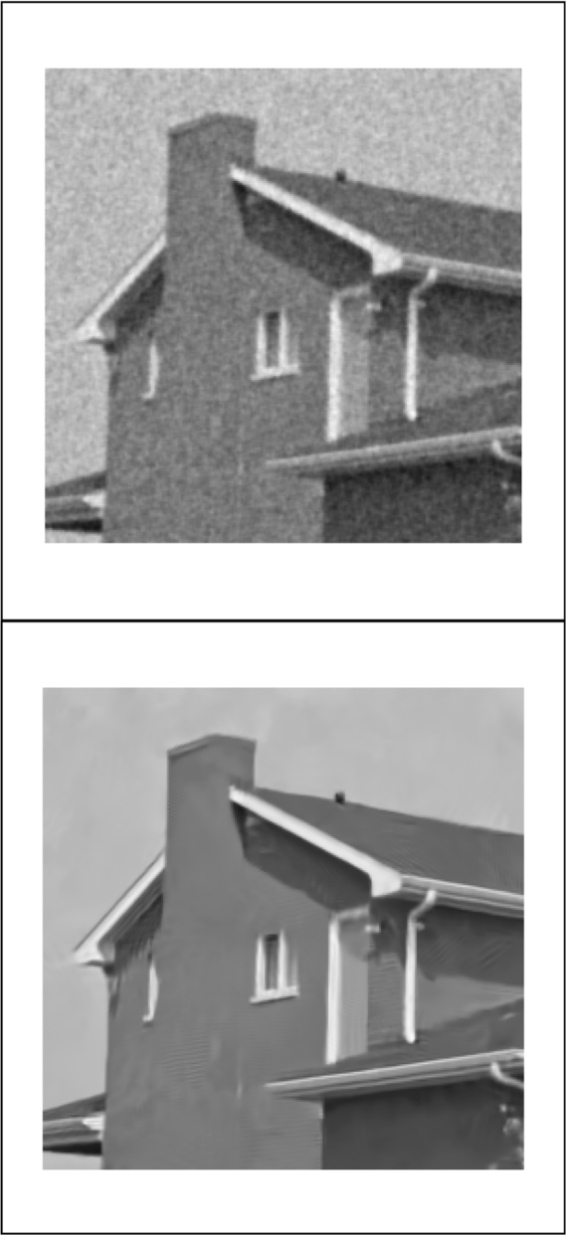
**Results of our method (up) and BM3D technique (down) with SNR = 25 for House.**

**Figure 15:**
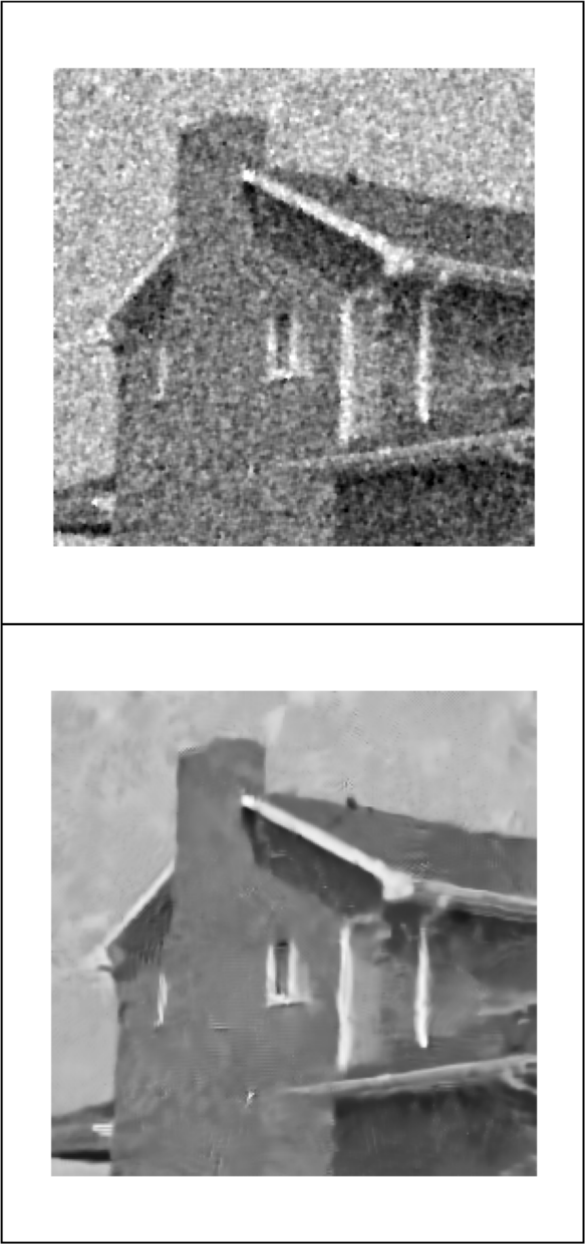
**Results of our method (up) and BM3D technique (down) with SNR = 75 for House.**

**Figure 16:**
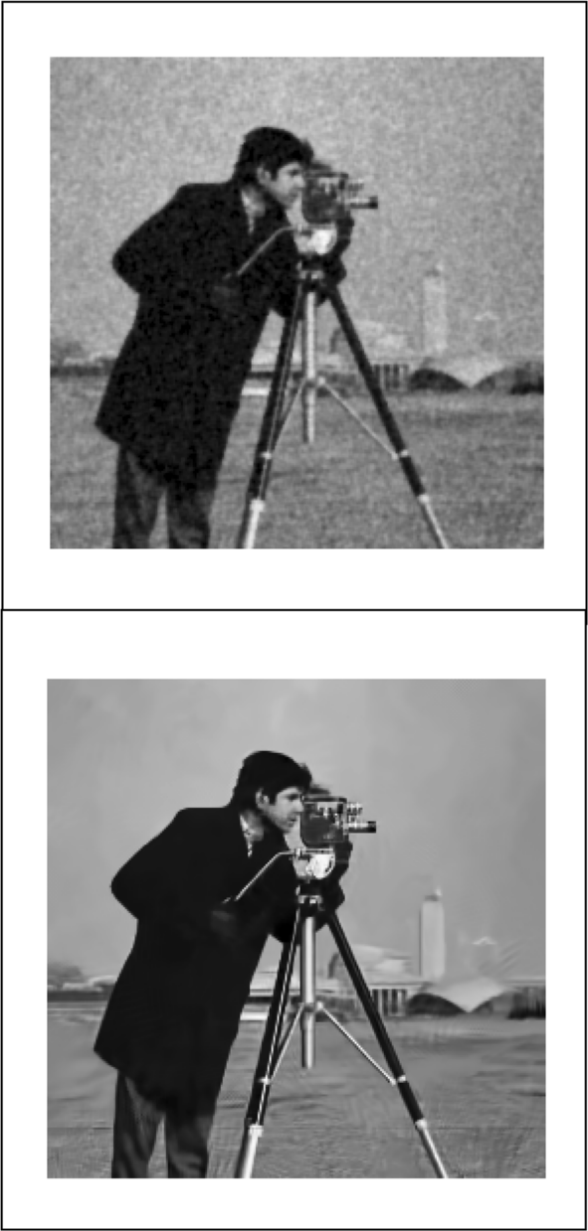
**Results of our method (up) and BM3D technique (down) with SNR = 25 for Cameraman.**

**Figure 17:**
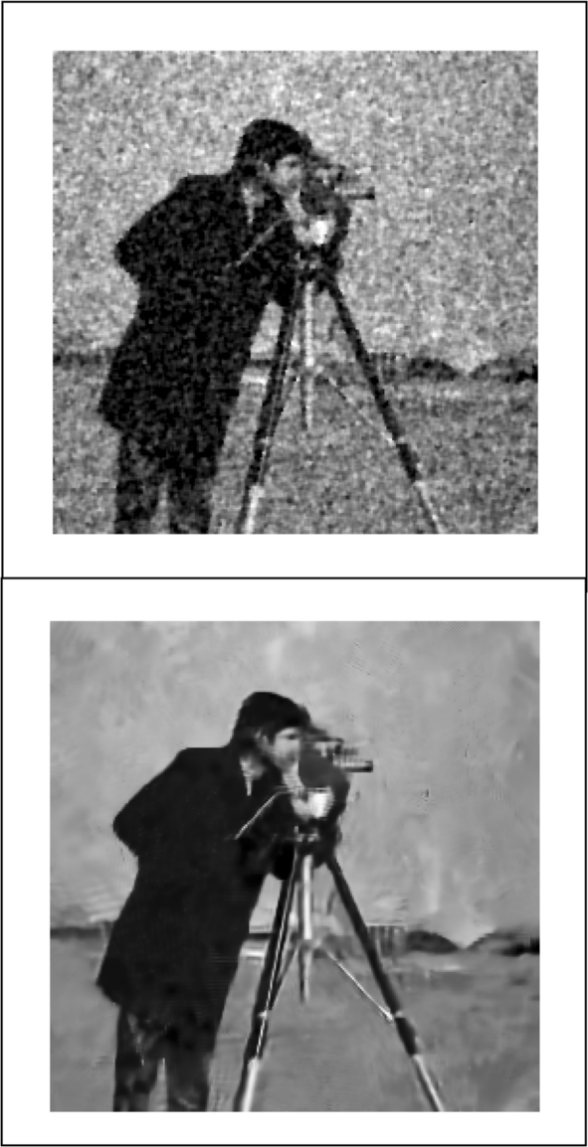
**Results of our method (up) and BM3D technique (down) with SNR = 75 for Cameraman.**

We implemented our method in two noisy conditions (0.005 and 0.1 noise variance using imnoise matlab code for Gaussian noise) and compared it with ROF model using evolve2D code with 10 iterations and Wiener filter using wiener2 (‘image’, [3 3]) matlab code. *f*(*u*_0_) can be interpreted as a noise intensity pattern in the image plane. Similar to ROF model in which $\left\Vert div\left(\frac{\nabla {u}_0}{\left|\nabla {u}_0\right|}\right)\right\Vert$ can be used as a measure of noise variance (Dabov et al. 2007), ‖*f*(*u*_0_)‖ is related to noise intensity. An example is shown in Figure 4. The results including noisy and denoised images are demonstrated in Figure 5 for Boat, Figure 6 for Man and Figure 7 for House. Figures 8, 9 and 10 show the results for Cameraman, Lena and Barbara respectively. TV for noisy and denoised images is shown in Tables 1 and 2. TV of the denoised image is totally comparable with ROF model. The computation time of our model is equal to ROF method with 10 iterations. *f*(*u*_0_) has a decreasing behavior during the filtring time. At start, *f*(*u*_0_) is high in noisy pixels identified by *SWN*. Because image intensity is reduced by Laplacian value, *f*(*u*_0_) tends to zero after some iteration. An example of such response is shown if Figure 11. Number of iterations is the only parameter that controls smoothness and running time. Large number of iteration makes the image blurry. Since *f*(*u*_0_) goes to zero after some iteration, we can implement the algorithm adaptively until *f*(*u*_0_) reaches a small value. This value should be estimated experimentally to make the algorithm applicable for every noisy condition. We also compared our method with BM3D technique for Boat, House and Cameraman in two SNR 25 and 75. The results are shown in Figures 12, 13, 14, 15, 16, 17. The denoising technique used in this work can be generalized to other applications for reducing any feature of the image (*F*(*u*)) in other image processing tasks such as deblurring. A proposing framework can be written as the following energy functional:

**Table 1:** **TV for 6 noisy and denoised images with noise variance 0.005**

**Noise variance (0.005)**	**TV (original mage)**	**TV (noisy image)**	**TV (ROF model)**	**TV (Wiener filter)**	**TV (our method)**
Boat	1.6	2.09	1.31	1.42	1.22
Man	1.76	2.20	1.32	1.51	1.22
House	1.3	1.81	1.21	1.27	1.14
Cameraman	1.67	2.13	1.33	1.53	1.20
Lena	1.26	1.74	1.16	1.24	1.12
Barbara	1.57	2.07	1.15	1.36	1.10

**Table 2:** **TV for 6 noisy and denoised images with noise variance 0.1**

**Noise variance (0.1)**	**TV (original image)**	**TV(noisy image)**	**TV (ROF model)**	**TV (Wiener filter)**	**TV (our method)**
Boat	1.6	8.68	1.63	2.20	1.43
Man	1.76	8.28	1.62	2.26	1.42
House	1.3	8.72	1.59	2.12	1.39
Cameraman	1.67	8.44	1.61	2.22	1.41
Lena	1.26	8.11	1.53	2.12	1.34
Barbara	1.57	8.50	1.54	2.14	1.35

19 $$ J={\displaystyle \iint }{\left(u-{u}_0\right)}^2+\lambda {\displaystyle \iint } Detector\left(F(u)\right) Wiegth\left(F(u)\right) $$

Such that:

$$ \left| Detector(F)\right|\le 1\kern0.75em  and\kern1em \left| Wiegth(F)\right|\le 1 $$

and *λ* is a control parameter determined experimentally. Minimizing the energy functional results the final relation for implementation.

20 $$ \frac{\partial J}{\partial u}={\displaystyle \iint }2\left(u-u0\right)+{\displaystyle \iint}\lambda \kern0.5em \frac{\partial (F)}{\partial (u)}\kern0.5em \left[\frac{\partial (Detector)}{\partial (F)} Wiegth(F)+\frac{\partial (Wiegth)}{\partial (F)} Detector(F)\right]=0 $$

As an example, for deblurring, *Detector*(*F*) and *Wiegth*(*F*) can be suggested as:

21 $$ Detector(F)=H\left({g}_x\ {g}_{x+\Delta x}\right)H\left({g}_y\ {g}_{y+\Delta y}\right) $$

22 $$ Wiegth(F)={e}^{-{\left({g}_x-{g}_{x+\Delta x}\right)}^2}{e}^{-{\left({g}_y-{g}_{y+\Delta y}\right)}^2} $$

where the blurring locations identified when the sign of change in intensity of two adjacent pixels is equal (*g*_*x*_ *g*_*x* + Δ*x*_ > 0 , *g*_*y*_ *g*_*y* + Δ*y*_ > 0), and weighted by difference in their gradient components for x and y directions. Computing $\frac{\partial J}{\partial u}=0$ the final relation is:

23 $$ u={u}_0+\lambda \left({u}_{0 xxxx}+{u}_{0 yyyy}\right)H\left({g}_x\ {g}_{x+\Delta x}\right)H\left({g}_y\ {g}_{y+\Delta y}\right){e}^{-{\left({g}_x-{g}_{x+\Delta x}\right)}^2}{e}^{-{\left({g}_y-{g}_{y+\Delta y}\right)}^2} $$

This relation shows that in the blurred locations, identified by the blurring detector, *u* is restored by the forth order of differentiation in the blurred areas.

## Conclusion

We presented a noise removal method using a local switching noise estimator in an energy functional minimizing process. We showed that in addition to using Laplacian in edge detection tasks, it can be used for noise removal applications. Smoothness can be easily controlled just by the number of iterations. We compared the method with some classic methods like ROF and Wiener filters based on TV value and also with the state-of-the-art BM3D. The result of denoising is totally comparable with ROF model. The computation time is equal to ROF model with 10 iterations. Based on experimental results we concluded that in relation to classic filters like ROF the method appears to be easy, fast and applicable for many noisy images. We analyzed that the technique can be applied for other image processing applications like deblurring by defining the appropriate detector and weight functions. The main disadvantage of the method is its filtering action on the area of the image including low intensity edges. The method can be improved to represent better response by defining better noise detectors.

## Appendix A: driving denoising equation

Here we drive the denoising equation by minimizing the energy functional. First we use Taylor series and relate *u*(*x* − Δ*x*, *y*) to x component of the gradient of the image *u*_*x*_. We have:

24 $$ u\left(x-\Delta x,y\right)=u\left(x,y\right)-\Delta x\ {u}_x+\frac{1}{2}\Delta {x}^2\ {u}_{xx} $$

Then *g*_*x*_ can be computed as:

25 $$ {g}_x = {u}_x-\frac{1}{2}\Delta x\ {u}_{xx} $$

And for *g*_*x* + Δ*x*_ we can write:

26 $$ {g}_{x+\Delta x} = {u}_x+\frac{1}{2}\Delta x\ {u}_{xx} $$

Using Taylor series similarly for y components, *g*_*y*_ and *g*_*y* + Δ*y*_ are:

27 $$ {g}_y = {u}_y-\frac{1}{2}\Delta y\ {u}_{yy} $$

28 $$ {g}_{y+\Delta y} = {u}_y+\frac{1}{2}\Delta y\ {u}_{yy} $$

Based on the above relations, we write:

29 $$ -{g}_x\ {g}_{x+\Delta x}= - {u_x}^2+\frac{1}{4}\Delta {x}^2\ {u_{xx}}^2 $$

30 $$ -{g}_y\ {g}_{y+\Delta y}= - {u_y}^2+\frac{1}{4}\Delta {y}^2\ {u_{yy}}^2 $$

The minimizer *u* is found by $\frac{\partial J}{\partial u}=0$. Considering the following relations for simplicity:

31 $$ {H}_1=H\left(-{g}_x\ {g}_{x+\Delta x}\right)\kern0.75em ,\kern1.25em {H}_2=H\left(-{g}_y\ {g}_{y+\Delta y}\right)\kern1em ,\kern1.25em {Z}_1={e}^{-{\left({g}_x+{g}_{x+\Delta x}\right)}^2}\kern0.75em ,\kern1em {Z}_2={e}^{-{\left({g}_y+{g}_{y+\Delta y}\right)}^2} $$

We can write:

32 $$ J={\displaystyle \iint }{\left(u-{u}_0\right)}^2+\lambda {\displaystyle \iint }{H}_1\ {H}_2\ {Z}_1\ {Z}_2 $$

Differentiating *J* with respect to *u* we have:

33 $$ \begin{array}{l}\frac{\partial J}{\partial u}={\displaystyle \iint}\Big[2\left(u-{u}_0\right)+\frac{\partial \lambda }{\partial u}\left({H}_1\ {H}_2{Z}_1\ {Z}_2\right)+\lambda \frac{\partial H\left(-{g}_x\ {g}_{x+\Delta x}\right)}{\partial u}{H}_2{Z}_1\ {Z}_2\\ {}+\lambda \frac{\partial H\left(-{g}_y\ {g}_{y+\Delta y}\right)}{\partial u}{H}_1{Z}_1\ {Z}_2-2\lambda \left({g}_x+{g}_{x+\Delta x}\right)\frac{\partial \left({g}_x+{g}_{x+\Delta x}\right)}{\partial u}{H}_1\ {H}_2{Z}_1\ {Z}_2\\ {}-2\lambda \left({g}_y+{g}_{y+\Delta y}\right)\frac{\partial \left({g}_y+{g}_{y+\Delta y}\right)}{\partial u}{H}_1\ {H}_2{Z}_1\ {Z}_2\Big]=0\end{array} $$

First we compute $\frac{\partial {g}_x}{\partial u}$:

34 $$ \frac{\partial {g}_x}{\partial u}=\left[\frac{u_{xx}}{u_x}+\frac{u_{xy}}{u_y}\right]-\left[\frac{\Delta x}{2}\left(\ \frac{u_{xxx}}{u_x}+\frac{u_{xxy}}{u_y}\right)\right] = {\gamma}_x-{\beta}_x $$

Similarly for *g*_*x* + Δ*x*_ we have:

35 $$ \frac{\partial {g}_{x+\Delta x}}{\partial u}={\gamma}_x+{\beta}_x $$

Then we can write:

36 $$ \frac{\partial \left({g}_x+{g}_{x+\Delta x}\right)}{\partial u}=2{\gamma}_x=2\left[\frac{u_{xx}}{u_x}+\frac{u_{xy}}{u_y}\right] $$

And for y direction:

37 $$ \frac{\partial \left({g}_y+{g}_{y+\Delta y}\right)}{\partial u}=2{\gamma}_y=2\left[\frac{u_{yy}}{u_y}+\frac{u_{yx}}{u_x}\right] $$

Second we have:

38 $$ \frac{\partial \left(-{g}_x\ {g}_{x+\Delta x}\right)}{\partial u}=-2{u}_{xx}-2{u}_{xy}\frac{u_x}{u_y}+\frac{\Delta {x}^2}{2}\left(\frac{u_{xxx}}{u_x}+\frac{u_{xxy}}{u_y}\right){u}_{xx} $$

39 $$ \frac{\partial \left(-{g}_y\ {g}_{y+\Delta y}\right)}{\partial u}=-2{u}_{yy}-2{u}_{yx}\frac{u_y}{u_x}+\frac{\Delta {y}^2}{2}\left(\frac{u_{yyy}}{u_y}+\frac{u_{yyx}}{u_x}\right){u}_{yy} $$

Derivative of Heaviside Function appears the impulse function:

40 $$ \frac{\partial H(.)}{\partial u}=\frac{\partial H(.)}{\partial (.)}\ \frac{\partial (.)}{\partial u}=\delta (.)\ \frac{\partial (.)}{\partial u} $$

where *δ*(.) is the impulse function. We Ignore its effect in the denoising process and assume *λ* is constant during the implementation. Therefore we have:

41 $$ \frac{\partial H\left(-{g}_x\ {g}_{x+\Delta x}\right)}{\partial u}\approx 0\kern1.25em ,\kern1.75em \frac{\partial H\left(-{g}_y\ {g}_{y+\Delta y}\right)}{\partial u}\approx 0\kern1.75em ,\kern1.25em \frac{\partial \lambda }{\partial u}\approx 0 $$

Then $\frac{\partial J}{\partial u}$ can be simplified to:

42 $$ \frac{\partial J}{\partial u}={\displaystyle \iint }2\left(u-u0\right)-4\lambda {\displaystyle \iint }{H}_1\ {H}_2{Z}_1\ {Z}_2\left[\left({g}_x+{g}_{x+\Delta x}\right)\left(\frac{u_{xx}}{u_x}+\frac{u_{xy}}{u_y}\right)+\left({g}_y+{g}_{y+\Delta y}\right)\left(\frac{u_{yy}}{u_y}+\frac{u_{yx}}{u_x}\right)\right]=0 $$

After simplification, the result is:

43 $$ u={u}_0+4\lambda\ SWN\ \left(\nabla {u}_x\ {\gamma}_x+\nabla {u}_y\ {\gamma}_y\right)={u}_0+4\lambda\ SWN\ \left(\ {\nabla}^2u + \nabla {u}^2\frac{u_{xy}}{u_x{u}_y}\right) $$

Because *u*_*x*_*u*_*y*_ in the second part of the equation may create zero in the denominator, we write:

44 $$ \nabla {u}^2\frac{u_{xy}}{u_x{u}_y}={u_x}^2\frac{u_{xy}}{u_x{u}_y}+{u_y}^2\frac{u_{xy}}{u_x{u}_y} $$

Using partial differential relations we have:

45 $$ {u_x}^2\frac{u_{xy}}{u_x{u}_y} = \frac{{\left(\frac{\partial u\ }{\partial x}\right)}^2}{\frac{\partial u\ \partial u}{\partial x\partial y}}\ \frac{\partial^2u}{\partial x\partial y}=\frac{\partial^2u}{\partial {x}^2}={u}_{xx} $$

Similarly for y component:

46 $$ {u_y}^2\frac{u_{xy}}{u_x{u}_y} = \frac{{\left(\frac{\partial u\ }{\partial y}\right)}^2}{\frac{\partial u\ \partial u}{\partial x\partial y}}\ \frac{\partial^2u}{\partial x\partial y}=\frac{\partial^2u}{\partial {y}^2}={u}_{yy} $$

The final equation is:

47 $$ u={u}_0+2\lambda\ SWN\kern0.5em {\nabla}^2u = {u}_0+\lambda\ f(u) $$
